# Cloning and Functional Characterization of *PmCPS*, a Copalyl Diphosphate Synthase Gene from *Pinus massoniana* Lamb.

**DOI:** 10.3390/plants15213273

**Published:** 2026-10-23

**Authors:** Jing Liu, Pengzhou Liu, Dengbao Wang, Wenjuan Su, Chengcheng Wu, Guangtong Cui, Kongshu Ji

**Affiliations:** 1 State Key Laboratory of Tree Genetics and Breeding, Nanjing Forestry University, Nanjing 210037, China; jingliu@njfu.edu.cn (J.L.); lpz9527@njfu.edu.cn (P.L.); suwenj@njfu.edu.cn (W.S.); ciciwoow@163.com (C.W.); 3240100006@njfu.edu.cn (G.C.); 2 Co-Innovation Center for Sustainable Forestry in Southern China, Nanjing Forestry University, Nanjing 210037, China; 3 The Key Laboratory of National Forestry and Grassland Administration for Tropical Forestry Research, Research Institute of Tropical Forestry, Chinese Academy of Forestry, Guangzhou 510520, China; dbwtact@163.com

**Keywords:** diterpenoid biosynthesis, gibberellin, virus-induced gene silencing, heterologous expression

## Abstract

*Pinus massoniana* Lamb. is an important timber- and oleoresin-producing tree species in southern China. Copalyl diphosphate synthase (CPS) catalyzes the cyclization of geranylgeranyl diphosphate (GGPP) to copalyl diphosphate (CPP), representing a key step in the biosynthesis of gibberellins (GAs) and diverse diterpenoids. In this study, *PmCPS* was cloned from *P. massoniana*, and its protein structure, phylogenetic relationships, tissue-specific expression pattern, and promoter cis-acting elements were characterized. Its biological function was further investigated using virus-induced gene silencing (VIGS) in *P. massoniana* and heterologous overexpression in *Arabidopsis thaliana*. PmCPS exhibited the characteristic structural features of plant diterpene synthases. *PmCPS* transcripts were most abundant in needles, reaching approximately 90 times the level in female strobili, and their abundance increased significantly to about 1.7 times the control level 3 h after GA_3_ treatment. Silencing *PmCPS* reduced CPS enzymatic activity from approximately 16.8 to 12.0 IU g^−1^ and decreased GA_3_ content from approximately 57 to 46 ng g^−1^, and downregulated genes associated with the 2-C-methyl-D-erythritol 4-phosphate (MEP) pathway and GA biosynthesis. Conversely, heterologous expression of *PmCPS* increased CPS activity to 1.60–1.98 times the wild-type level and endogenous GA_3_ content from 43.90 to 71.50–80.39 ng g^−1^ in *A. thaliana*, accumulated more than twice the fresh weight of wild-type plants, and significantly accelerated bolting. Under abscisic acid (ABA), methyl jasmonate (MeJA), NaCl, and D-mannitol treatments, the transgenic plants maintained greater primary root growth than wild-type plants. Collectively, these findings indicate that *PmCPS* is a key component of GA-related diterpenoid metabolism in *P. massoniana* and may contribute to plant growth, development, and treatment responses by modulating plastidial isoprenoid metabolism and phytohormone homeostasis. This study advances our understanding of diterpenoid metabolism and hormonal regulation in *P. massoniana* and identifies *PmCPS* as a promising candidate gene for molecular breeding targeting growth- and stress-related traits.

## 1. Introduction

*Pinus massoniana* Lamb. is an important native evergreen conifer that is widely used for afforestation in southern China and provides multiple benefits, including soil and water conservation, ecological restoration, timber production, and oleoresin utilization [1,2]. As one of the principal oleoresin-producing tree species in China, *P. massoniana* accounts for a substantial proportion of the country’s pine oleoresin production [2]. Pine oleoresin consists primarily of diterpenoid resin acids and volatile components dominated by monoterpenes and sesquiterpenes. It serves not only as an important industrial raw material but also as a natural chemical defense against insect herbivory and pathogen invasion [3,4,5,6]. Terpenoids such as α-pinene, β-pinene, limonene, and longifolene have been associated with reduced survival of the pine wood nematode and enhanced host resistance [6]. Recent metabolomic work identified 7-dehydrocholesterol as a candidate early diagnostic indicator following *Bursaphelenchus xylophilus* infection in *P. massoniana* [7], highlighting the value of metabolite profiling for understanding pine–nematode interactions. In recent years, genetic improvement research on *P. massoniana* has focused primarily on photosynthesis, root development and phytohormone signaling, terpenoid metabolism, cellulose and lignin biosynthesis, and responses to biotic and abiotic stresses [1,8,9,10,11]. Gibberellins (GAs) are diterpenoid phytohormones that regulate seed germination, stem elongation, vascular development, and other physiological processes and interact extensively with terpenoid metabolism and plant defense signaling [12,13]. Therefore, elucidating the functions of key genes involved in diterpenoid metabolism is essential for understanding the mechanisms underlying growth and development, oleoresin formation, and stress adaptation in *P. massoniana*.

Diterpenoids are C_20_ natural products derived from four isoprene units and exhibit remarkable structural and functional diversity, particularly in gymnosperms such as members of the Pinaceae [4,14]. Plant diterpenoids include GAs, which regulate growth and development, as well as specialized metabolites with defensive functions, including resin acids and phytoalexins [3,4,15,16]. The plastidial 2-C-methyl-D-erythritol 4-phosphate (MEP) pathway supplies isopentenyl diphosphate (IPP) and dimethylallyl diphosphate (DMAPP) for diterpenoid biosynthesis. These precursors are subsequently converted to geranylgeranyl diphosphate (GGPP) by geranylgeranyl diphosphate synthase [17,18]. GGPP then undergoes cyclization and structural rearrangement catalyzed by diterpene synthases (diTPSs), giving rise to diverse diterpenoid scaffolds [19,20,21]. Copalyl diphosphate synthase (CPS) is a class II diterpene cyclase that typically contains the conserved DxDD catalytic motif and initiates GGPP cyclization through protonation, thereby producing copalyl diphosphate (CPP) with a specific stereochemical configuration [20,21,22]. Three principal stereoisomers of CPP have been identified: (+)-CPP, *ent*-CPP, and *syn*-CPP [15,16,23,24,25]. As a key enzyme in the biosynthesis of GAs and numerous defensive diterpenoids, CPS occupies a crucial branch point in diterpenoid metabolism; therefore, its catalytic properties and expression levels can directly influence the biosynthesis and accumulation of different diterpenoid products [12,21].

The functions of *CPS* genes have been characterized in diverse plant lineages, and their evolutionary divergence and regulatory mechanisms are important areas of research in plant diterpenoid metabolism. Some diterpene synthases in bryophytes and other early-diverging land plants possess both CPS and *ent*-kaurene synthase (KS) activities [26]. In seed-plant GA biosynthesis, *CPS* and *KS* generally evolved as separate monofunctional enzymes in gymnosperms and angiosperms; however, conifer specialized metabolism includes both monofunctional and bifunctional diTPSs [19,22,27]. This evolutionary transition provided an important foundation for the functional diversification of plant diterpenoid metabolism [19,26]. In *Arabidopsis thaliana*, foxtail millet, and other plant species, loss-of-function mutations in *CPS* genes involved in GA biosynthesis generally result in characteristic GA-deficient phenotypes, including dwarfism, abnormal organ development, and impaired reproductive development, demonstrating the essential role of *CPS* in vegetative and reproductive growth [28,29]. Furthermore, certain *CPS* family members in rice, *Salvia miltiorrhiza*, *Tripterygium wilfordii*, *Isodon rubescens*, and *Andrographis paniculata* participate in the biosynthesis of phytoalexins or pharmacologically active diterpenoids. These findings indicate that the *CPS* gene family has undergone functional divergence, with different members contributing to either primary GA metabolism or specialized defensive metabolism [15,16,23,24,25].

In conifers, diterpenoid resin acids are major constituents of defensive oleoresin, and CPS-mediated cyclization represents a critical step in the formation of their carbon skeletons [3,4,5]. Multiple functionally divergent CPS enzymes and CPS-related diTPSs have been identified in gymnosperms, including *P. glauca*, *P. contorta*, *P. banksiana*, *P. abies*, and *A. grandis*, indicating that gymnosperm diTPSs perform distinct catalytic functions in primary and defensive metabolism [22,27,30,31]. Genes encoding these diTPSs respond to insect herbivory, mechanical wounding, and methyl jasmonate (MeJA) treatment, thereby promoting traumatic resin duct formation and the biosynthesis and accumulation of defensive terpenoids [32,33,34,35]. Previous studies have also shown that the promoter regions of genes associated with terpenoid metabolism in *P. massoniana* contain diverse cis-acting elements related to MeJA, abscisic acid, light, and environmental stress responses, suggesting that terpenoid biosynthesis is coordinately regulated by phytohormone signaling, environmental stimuli, and transcription factors [8]. However, research on terpenoid metabolism in *P. massoniana* has focused predominantly on upstream precursor-supply genes, including *DXS*, *DXR*, and *GGPPS* [9,10,11], whereas the sequence characteristics, expression patterns, and biological functions of CPS, a key downstream branch-point enzyme, remain poorly understood. Whether *PmCPS* participates in GA homeostasis, defensive diterpenoid metabolism, and stress responses in *P. massoniana* therefore requires further investigation.

In this study, the full-length coding sequence of *PmCPS* was cloned from *P. massoniana*, and its protein structural characteristics, phylogenetic relationships, tissue-specific expression pattern, and promoter cis-acting elements were systematically characterized. The inducible expression pattern of *PmCPS* was then examined under phytohormone and abiotic stress treatments, and its biological functions were evaluated using virus-induced gene silencing (VIGS) in *P. massoniana* and heterologous overexpression in *A. thaliana*. This study aimed to clarify the roles of *PmCPS* in GA biosynthesis, terpenoid metabolic regulation, plant growth and development, and treatment responses. The findings provide a foundation for refining the diterpenoid regulatory network of *P. massoniana* and identify a candidate gene for the molecular breeding of growth- and stress-related traits.

## 2. Results

### 2.1. Bioinformatic Characterization of PmCPS

**Figure 1:**
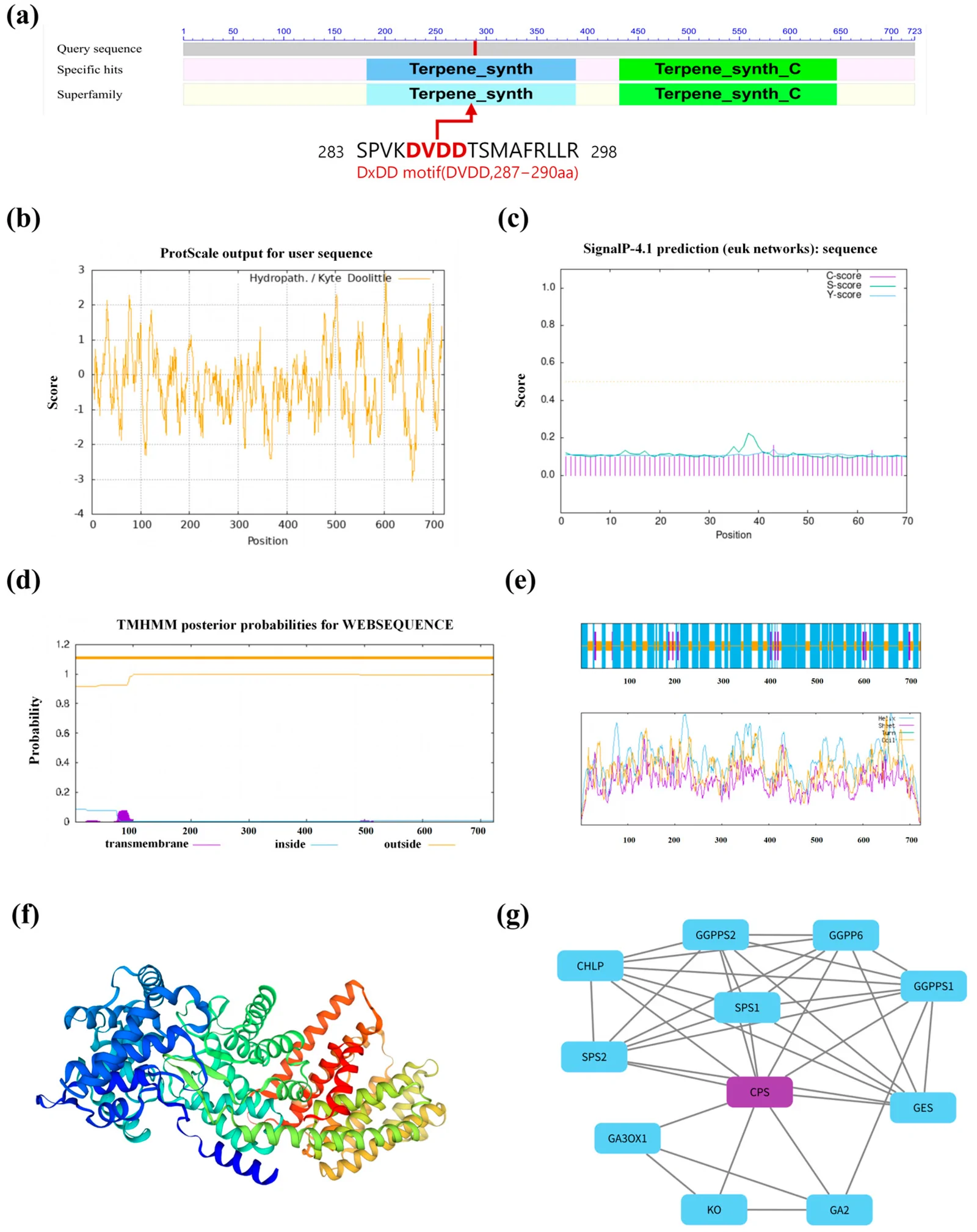
Bioinformatics analysis of the PmCPS protein. (**a**) Conserved-domain architecture of PmCPS; (**b**) protein hydropathy; (**c**) signal peptide prediction; (**d**) transmembrane helix prediction; (**e**) protein secondary structure; (**f**) three-dimensional homology model of PmCPS; and (**g**) CPS-centered protein association network constructed using *A. thaliana* homologs. Network edges represent database-predicted functional associations and do not necessarily indicate direct physical interactions between PmCPS and the associated proteins.

The full-length coding sequence of *PmCPS* was successfully cloned from *P. massoniana*. Its open reading frame (ORF) was 2283 bp long (Figure S1). Conserved-domain analysis revealed that PmCPS contained two characteristic terpene synthase domains: a Terpene_synth domain spanning amino acid residues 182–388 and a Terpene_synth_C domain spanning residues 432–646. These findings indicated that PmCPS belongs to the plant terpene synthase superfamily (Figure 1a).

ProtScale analysis showed that the hydropathy indices of the PmCPS amino acid sequence were predominantly negative, with a mean value of −0.260, indicating that PmCPS is predominantly hydrophilic (Figure 1b). No apparent signal peptide cleavage site was predicted by SignalP 4.1 (Figure 1c), and TMHMM analysis identified no reliable transmembrane helices (Figure 1d). Secondary structure prediction indicated that PmCPS was composed predominantly of α-helices and random coils, which accounted for 64.59% and 31.67% of the protein, respectively, whereas β-strands accounted for 3.73% (Figure 1e). The three-dimensional homology model, constructed using a CPS homolog from Chinese pine (*Pinus tabuliformis*) as the template, showed high confidence (Figure 1f). A STRING-based association network constructed using *A. thaliana* homologs placed CPS at the center and predicted associations with proteins involved in terpenoid scaffold biosynthesis, including GGPPS1, GGPPS2, GGPP6, CHLP, SPS1, SPS2, and GES, as well as proteins associated with GA metabolism, including KO, GA2, and GA3OX1 (Figure 1g).

### 2.2. Phylogenetic Relationships and Promoter Cis-Acting Elements of PmCPS

**Figure 2:**
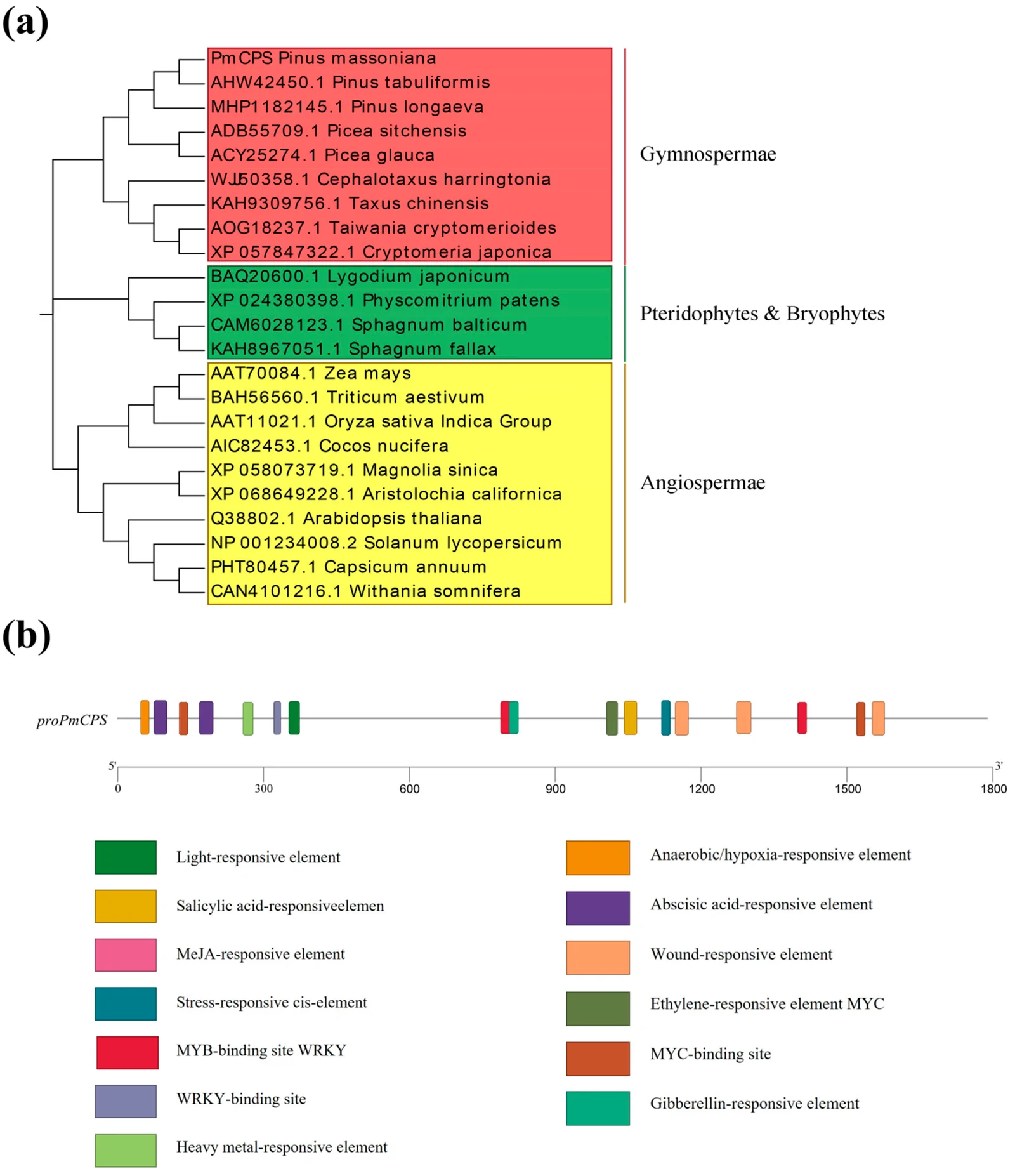
Phylogenetic relationships of PmCPS and cis-acting elements in the *PmCPS* promoter. (**a**) Phylogenetic analysis of PmCPS and CPS homologs from other plant species. The neighbor-joining tree was constructed with 1000 bootstrap replicates. Red, green, and yellow backgrounds indicate gymnosperms, ferns and bryophytes, and angiosperms, respectively; the PmCPS entry is placed at the top of the gymnosperm group. (**b**) Distribution of cis-acting elements within the 1718 bp sequence upstream of the *PmCPS* translation start site. Colors indicate elements associated with light responses, phytohormone responses, transcription factor binding, and environmental stress responses.

To investigate the evolutionary relationships of PmCPS, a phylogenetic tree was constructed using CPS homologs from 22 plant species. PmCPS formed a sister group with CPS from *P. tabuliformis*, indicating a close evolutionary relationship between the two proteins. PmCPS also formed a distinct clade with CPS homologs from other gymnosperms, including *P. longaeva*, *P. sitchensis*, *P. glauca*, *C. harringtonia*, *T. chinensis*, *T. cryptomerioides,* and *C. japonica.* CPS homologs from ferns and bryophytes occupied basal positions in the phylogenetic tree, whereas those from angiosperms, including maize, wheat, rice, *A. thaliana*, tomato, and pepper, clustered in a separate clade (Figure 2a).

Analysis of the 1718 bp sequence upstream of *PmCPS* identified numerous cis-acting elements associated with plant growth and environmental responses. These included elements responsive to light, MeJA, abscisic acid (ABA), salicylic acid (SA), gibberellins (GAs), and ethylene, as well as elements associated with anaerobic or hypoxic conditions, wounding, heavy-metal exposure, and general stress responses. Putative binding sites for transcription factors, including MYB, MYC, and WRKY proteins, were also distributed throughout the promoter region (Figure 2b).

### 2.3. Tissue-Specific Expression of PmCPS and Its Responses to Phytohormone and Stress Treatments

**Figure 3:**
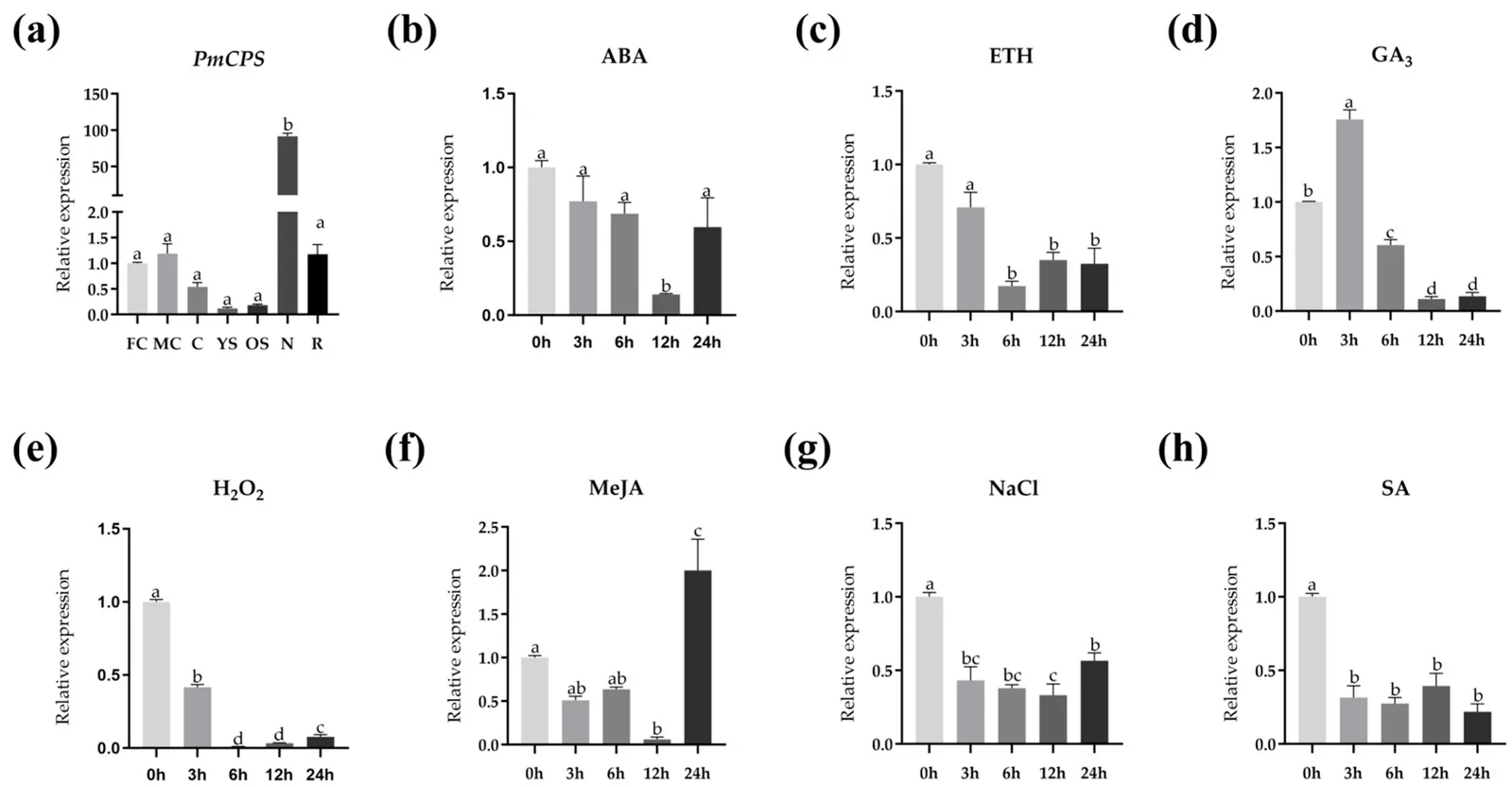
Tissue-specific expression pattern of *PmCPS* and its responses to phytohormone and abiotic stress treatments. (**a**) Relative expression levels of *PmCPS* in different tissues of *P. massoniana*; (**b**–**h**) relative expression levels of *PmCPS* in needles at 0, 3, 6, 12, and 24 h after ABA, ETH, GA_3_, H_2_O_2_, MeJA, NaCl, and SA treatments, respectively. FC, MC, C, YS, OS, N, and R denote female strobili, male strobili, cones, young stems, mature stems, needles, and roots, respectively. Female strobili (**a**) and the pretreatment 0 h samples (**b**–**h**) were used as calibrators. Data are presented as the mean ± standard error (SE) of three biological replicates (*n* = 3). Different lowercase letters indicate significant differences among tissues or treatment times, as determined by one-way analysis of variance followed by Duncan’s multiple range test (*p* < 0.05).

RT-qPCR analysis showed that *PmCPS* was expressed in all seven tissues examined in *P. massoniana*, although transcript abundance varied markedly among tissues. *PmCPS* transcript abundance was highest in needles and was significantly greater than that in male strobili, female strobili, cones, young stems, mature stems, and roots. Transcript abundance in needles was approximately 90 times that in female strobili (Figure 3a).

Phytohormone and abiotic stress treatments exerted distinct effects on *PmCPS* expression. Following ABA treatment, *PmCPS* expression decreased significantly only at 12 h (Figure 3b). Under ETH treatment, transcript abundance from 6 to 24 h was significantly lower than that at 0 h (Figure 3c). GA_3_ treatment significantly and transiently increased *PmCPS* expression to approximately 1.7 times the 0 h level at 3 h, after which expression declined rapidly (Figure 3d). H_2_O_2_ treatment significantly suppressed *PmCPS* expression, with the lowest transcript levels observed at 6 and 12 h (Figure 3e). Following MeJA treatment, *PmCPS* expression initially decreased and then increased, reaching approximately twice the 0 h level at 24 h (Figure 3f). NaCl treatment suppressed *PmCPS* expression throughout the 3 to 24 h treatment period, with the lowest level detected at 12 h (Figure 3g). Similarly, SA treatment significantly reduced *PmCPS* expression (Figure 3h).

### 2.4. VIGS-Mediated Silencing of PmCPS and Changes in CPS Enzymatic Activity

**Figure 4:**
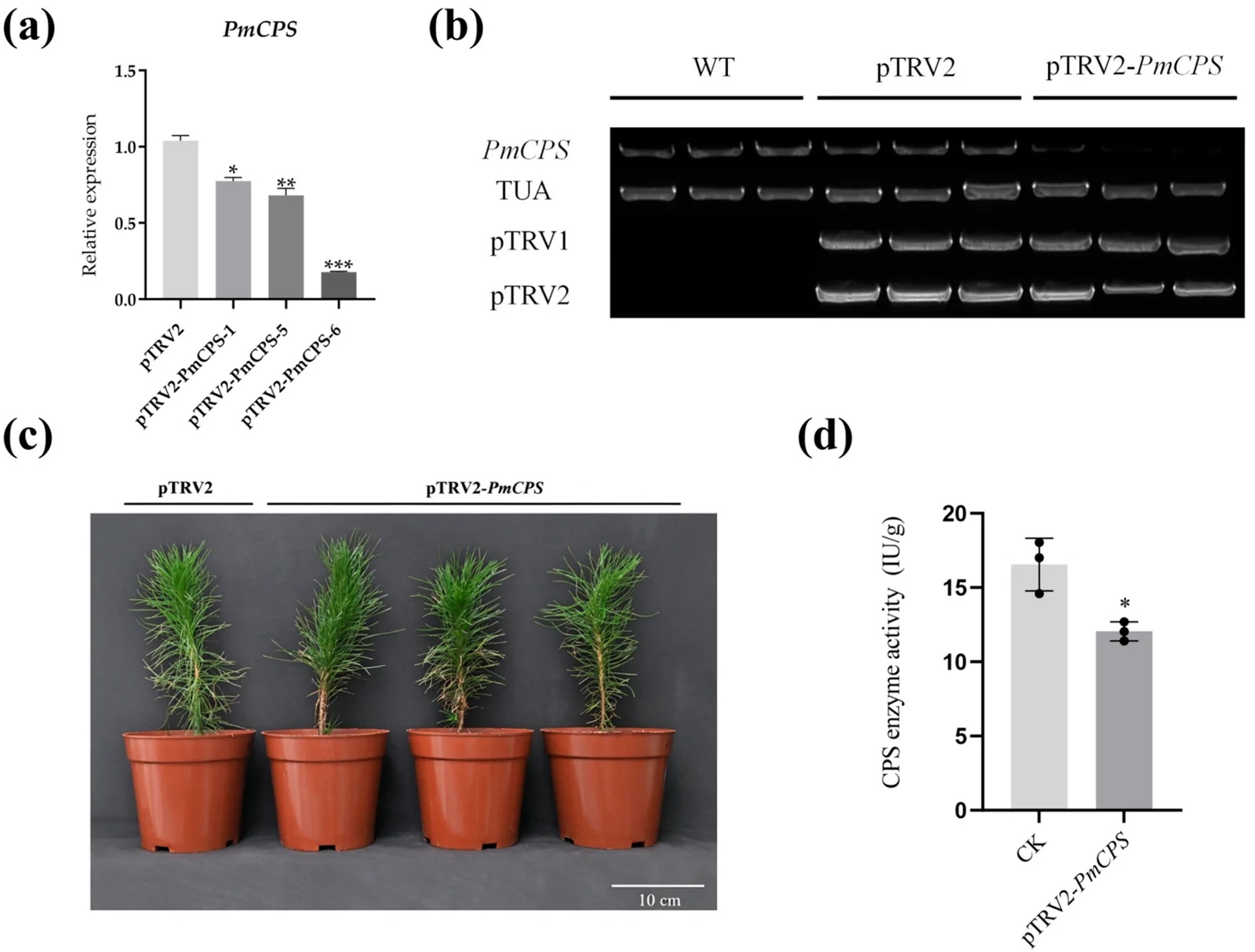
VIGS-mediated silencing of *PmCPS* in *Pinus massoniana* and its effect on CPS enzymatic activity. (**a**) Relative expression levels of *PmCPS* in the empty-vector control and three independently silenced plants; (**b**) semi-quantitative PCR analysis of *PmCPS* expression and the pTRV1 and pTRV2 viral vector components, with *TUA* used as the reference gene; (**c**) phenotypes of the pTRV2 empty-vector control and pTRV2-*PmCPS* plants; and (**d**) CPS enzymatic activity in the empty-vector control and *PmCPS*-silenced plants. WT, wild type; pTRV2/CK, pTRV1 + pTRV2 empty-vector control; pTRV2-*PmCPS*, *PmCPS*-silenced material (* *p* < 0.05; ** *p* < 0.01; *** *p* < 0.001; *n* = 3 biological replicates).

Compared with the empty-vector control (pTRV2), *PmCPS* transcript abundance in the pTRV2*-PmCPS*-1, pTRV2*-PmCPS*-5, and pTRV2*-PmCPS*-6 plants decreased to approximately 37%, 36%, and 21% of the control level, respectively. All reductions were highly significant, and the mean silencing efficiency was approximately 69% (Figure 4a). Semi-quantitative PCR analysis showed that the band intensity of the reference gene *TUA* was similar among the wild-type, empty-vector control, and silenced plants, whereas *PmCPS* bands were markedly weaker in the pTRV2-*PmCPS* plants. These results further confirmed effective *PmCPS* silencing (Figure 4b).

Phenotypic observations revealed no marked differences in overall growth between the pTRV2-*PmCPS* plants and the pTRV2 empty-vector control plants during the experimental period (Figure 4c). However, CPS enzymatic activity decreased significantly from approximately 16.6 IU g^−1^ in the empty-vector control (pTRV2) group to approximately 12.1 IU g^−1^ in the silenced plants (Figure 4d). This reduction was consistent with the decrease in *PmCPS* transcript abundance and further confirmed the effectiveness of VIGS-mediated silencing. These findings provide biochemical evidence that *PmCPS* participates in diterpenoid biosynthesis in *P. massoniana*.

### 2.5. Effects of PmCPS Silencing on Endogenous Phytohormone Levels and Pathway-Related Gene Expression

**Figure 5:**
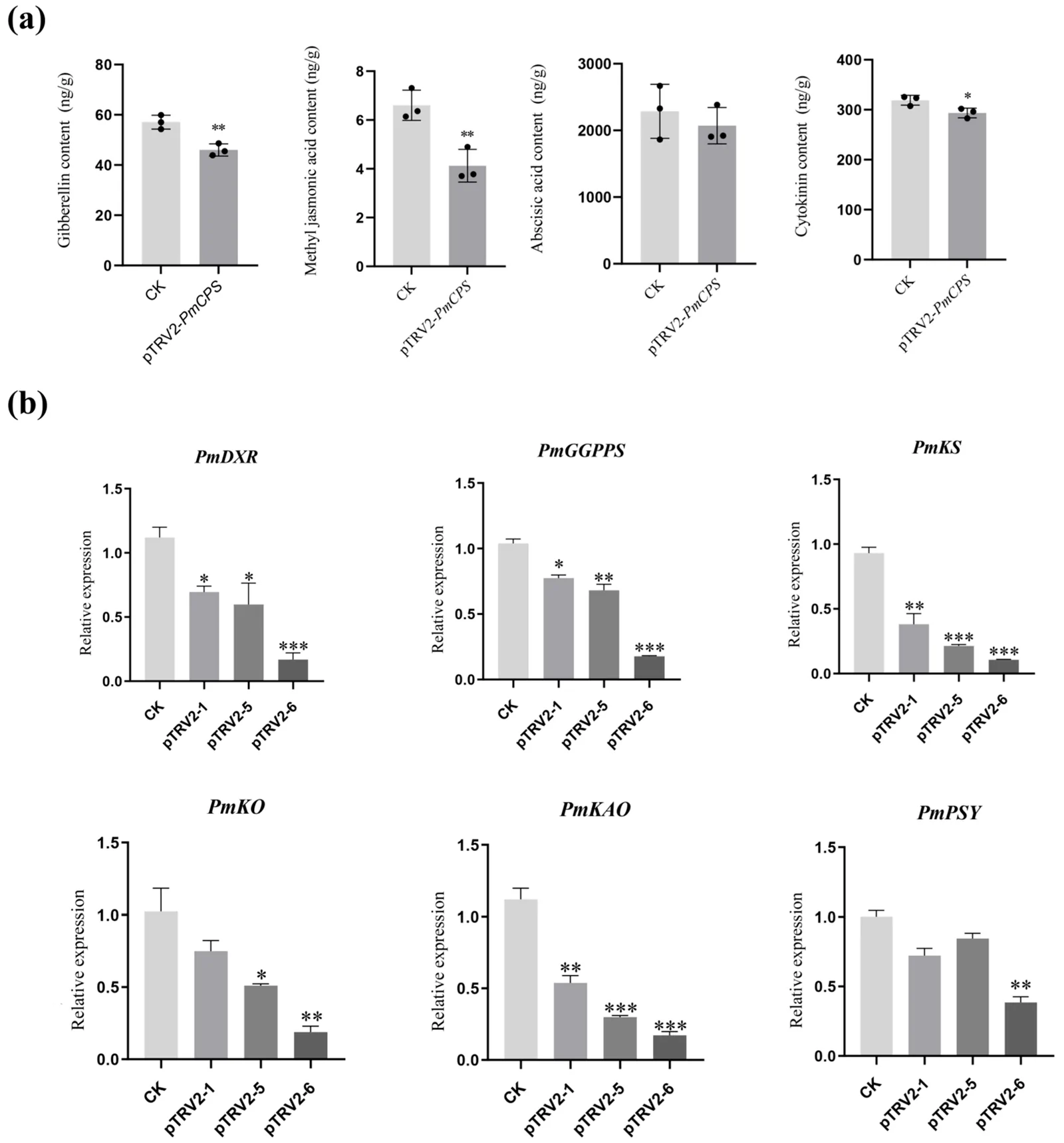
Effects of *PmCPS* silencing on endogenous phytohormone levels and the expression of diterpenoid metabolism-related genes. (**a**) GA_3_, MeJA, ABA, and CTK levels in the empty-vector control and *PmCPS*-silenced plants; (**b**) relative expression levels of *PmDXR*, *PmGGPPS*, *PmKS*, *PmKO*, *PmKAO*, and *PmPSY*. CK, pTRV1 + pTRV2 empty-vector control; pTRV2-1, pTRV2-5, and pTRV2-6, three independently generated *PmCPS*-silenced plants (* *p* < 0.05; ** *p* < 0.01; *** *p* < 0.001; *n* = 3 biological replicates).

To investigate whether *PmCPS* silencing was associated with phytohormone changes, endogenous GA_3_, MeJA, ABA, and cytokinin (CTK) levels were measured in silenced and control *P. massoniana* plants. Compared with the empty-vector control, GA_3_ content in pTRV2-*PmCPS* plants decreased from approximately 57 to 46 ng g^−1^, whereas MeJA content decreased from approximately 6.6 to 4.1 ng g^−1^; both reductions were significant at *p* < 0.01. CTK content also decreased significantly (*p* < 0.05), whereas ABA content decreased numerically but not significantly (Figure 5a). Thus, *PmCPS* silencing was accompanied by changes in the homeostasis of multiple phytohormones. The reduction in GA_3_ was consistent with the established role of CPS in GA biosynthesis, whereas the changes in MeJA, CTK, and ABA may reflect indirect metabolic feedback or phytohormone crosstalk. Collectively, these findings provide physiological evidence linking *PmCPS*-mediated diterpenoid metabolism with phytohormone homeostasis in *P. massoniana*.

Expression analysis of key genes in the MEP pathway and its downstream branches revealed broad transcriptional changes following *PmCPS* silencing. Compared with the control (CK), the precursor-supply genes *PmDXR* and *PmGGPPS* were significantly downregulated in all three independently silenced plants. The reductions were significant in pTRV2-1 and pTRV2-5 (*p* < 0.05) and were greatest in pTRV2-6, which showed the highest silencing efficiency (*p* < 0.001). Among the GA biosynthesis genes downstream of *PmCPS*, *PmKS*, *PmKO*, and *PmKAO* generally showed progressively lower expression as *PmCPS* silencing efficiency increased. *PmKS* was significantly downregulated in all silenced plants (*p* < 0.01 or *p* < 0.001). *PmKO* expression was significantly reduced in pTRV2-5 (*p* < 0.05) and pTRV2-6 (*p* < 0.01), whereas *PmKAO* was significantly downregulated in pTRV2-1 (*p* < 0.01) and showed greater reductions in pTRV2-5 and pTRV2-6 (*p* < 0.001). By contrast, *PmPSY*, a key carotenoid biosynthesis gene, was significantly downregulated only in pTRV2-6 (*p* < 0.01). Although decreasing trends were observed in the other two plants, the differences were not statistically significant (Figure 5b).

### 2.6. Generation and Growth Phenotypes of PmCPS-Overexpressing Arabidopsis thaliana

**Figure 6:**
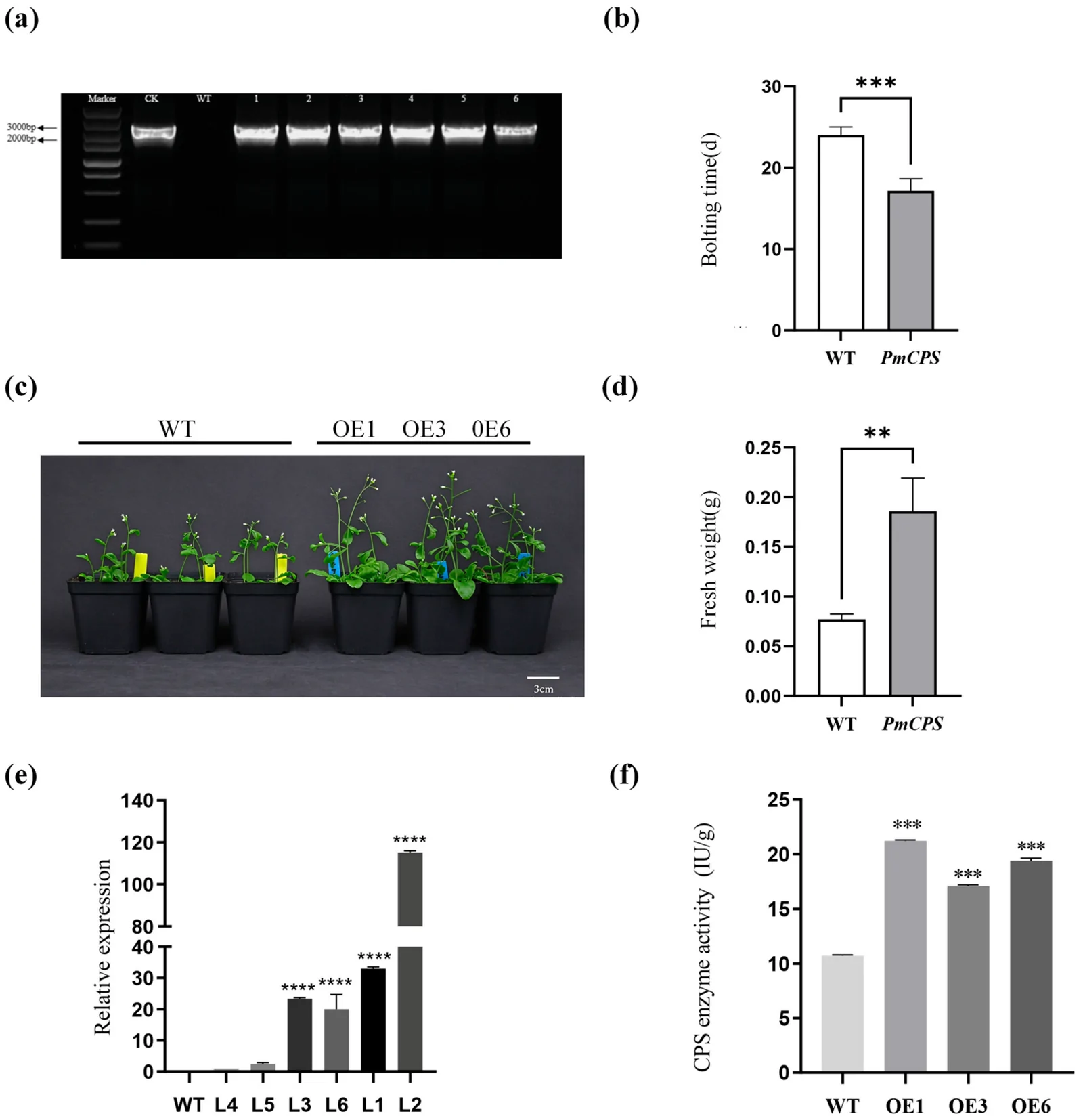
Generation, identification, and growth phenotyping of *PmCPS*-overexpressing *A. thaliana*. (**a**) Genomic PCR verification of *PmCPS*-overexpressing plants; (**b**) bolting time of wild-type and *PmCPS*-overexpressing plants; (**c**) growth phenotypes of wild-type and *PmCPS*-overexpressing plants (scale bar = 3 cm); (**d**) fresh weight of wild-type and *PmCPS*-overexpressing plants; (**e**) relative expression levels of *PmCPS* in different transgenic lines; (**f**) CPS enzymatic activity in wild-type plants and the OE1, OE3, and OE6 lines. In (**b**,**d**), *PmCPS* denotes the combined OE1, OE3, and OE6 group; individual lines are shown separately in (**f**). WT, wild type; CK, positive PCR control; 1–6, independent transgenic lines; OE, *PmCPS*-overexpressing line (** *p* < 0.01; *** *p* < 0.001; **** *p* < 0.0001; *n* = 3 biological replicates).

A *PmCPS*-specific amplicon was detected in the positive control (CK) and in genomic DNA from the six transgenic lines but not in wild-type (WT) plants, confirming the integration of *PmCPS* into the *A. thaliana* genome (Figure 6a).

Under identical growth conditions, *PmCPS*-overexpressing plants exhibited enhanced growth and accelerated development compared with WT plants. For the growth comparisons in Figure 6b–d, material from OE1, OE3, and OE6 was analyzed together as the *PmCPS* group. This combined group bolted significantly earlier (Figure 6b,c) and accumulated more than twice the fresh weight of WT plants (Figure 6d). RT-qPCR analysis of the six candidate lines L1–L6 revealed differences in *PmCPS* transcript abundance (Figure 6e). Following hygromycin-resistance segregation and successive-generation screening, three stable homozygous T_3_ lines, OE1, OE3, and OE6, were obtained from the original lines L1, L3, and L6, respectively (Figure 6e). CPS enzymatic activity was significantly higher in all three overexpression lines than in WT plants. OE1 showed the greatest increase, reaching 1.98 times the WT level, whereas CPS activities in OE6 and OE3 were 1.81 and 1.60 times the WT level, respectively (Figure 6f). Collectively, these results confirmed that *PmCPS* was stably expressed in three independent *A. thaliana* lines. The earlier bolting, increased fresh weight, and elevated CPS activity in these plants indicated that *PmCPS* overexpression was associated with accelerated growth and development. These lines were therefore used for subsequent analyses of GA metabolism and related growth traits.

### 2.7. Responses of PmCPS-Overexpressing Arabidopsis thaliana to Phytohormone and Abiotic Stress Treatments

**Figure 7:**
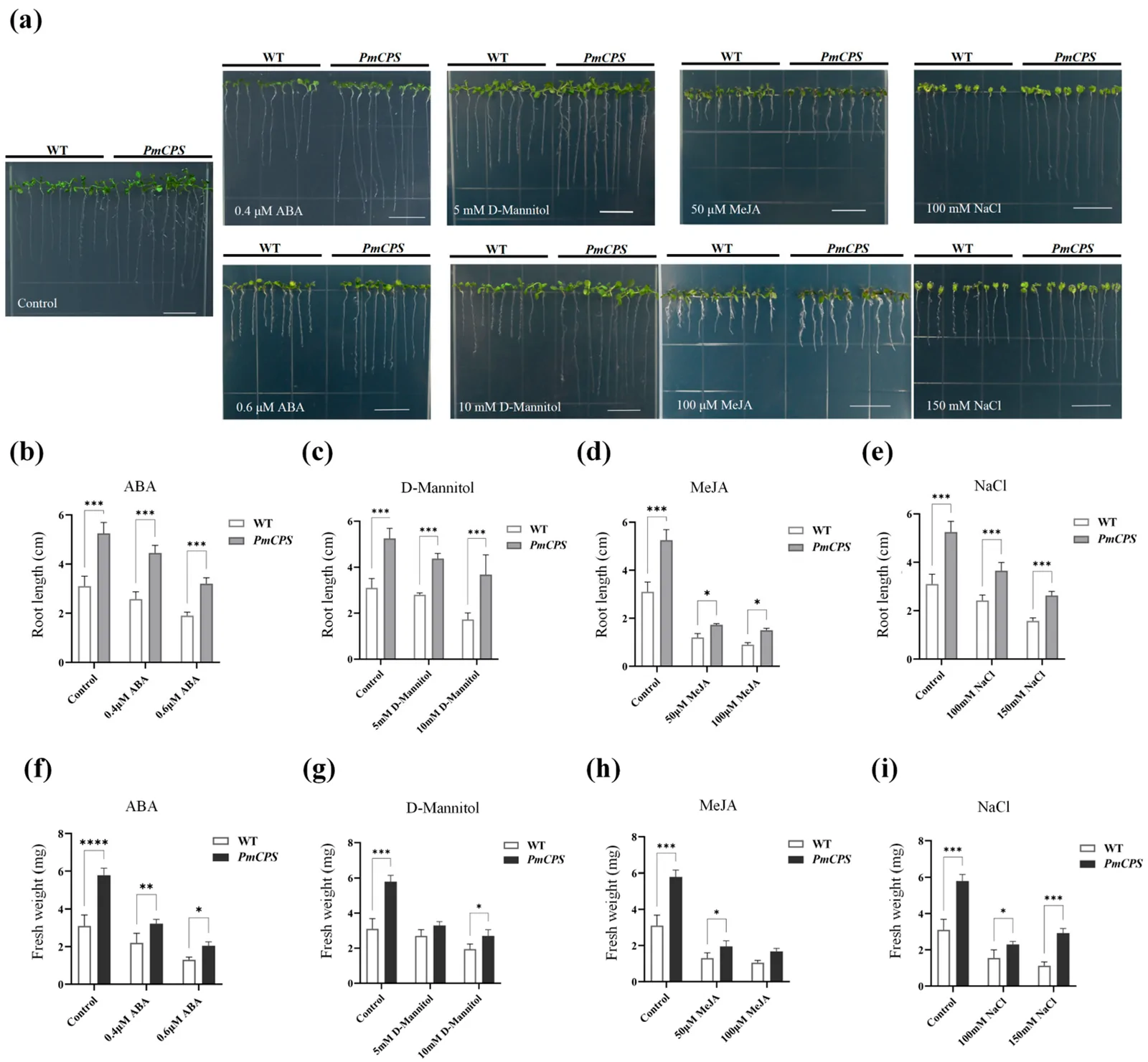
Growth phenotypes of *PmCPS*-overexpressing *A. thaliana* under different treatments. (**a**) Phenotypes of wild-type and *PmCPS*-overexpressing seedlings under control conditions and after treatment with ABA, D-mannitol, MeJA, or NaCl. (**b**–**e**) Primary root lengths following ABA, D-mannitol, MeJA, and NaCl treatments, respectively. (**f**–**i**) Fresh weights following the corresponding treatments. Treatment concentrations were 0.4 and 0.6 μM ABA, 5 and 10 mM D-mannitol, 50 and 100 μM MeJA, and 100 and 150 mM NaCl. WT, wild type; *PmCPS*, data combined from the independent T_3_ lines OE1, OE3, and OE6 (* *p* < 0.05; ** *p* < 0.01; *** *p* < 0.001; **** *p* < 0.0001; *n* = 3 biological replicates).

To investigate the effects of *PmCPS* overexpression on plant growth under phytohormone and abiotic stress treatments, primary root length and fresh weight were measured in wild-type plants and the combined T_3_ OE1, OE3, and OE6 group exposed to ABA, D-mannitol, MeJA, or NaCl. Compared with untreated controls, all treatments inhibited seedling growth to varying degrees. Overall, primary root length and fresh weight decreased as treatment concentrations increased (Figure 7a–i).

Under control conditions, the combined *PmCPS*-overexpressing group exhibited significantly greater primary root length and fresh weight than wild-type plants. Following treatment with 0.4 or 0.6 μM ABA, 5 or 10 mM D-mannitol, 50 or 100 μM MeJA, or 100 or 150 mM NaCl, the absolute primary root lengths of the combined overexpression group were significantly greater than those of wild-type plants under the corresponding conditions (Figure 7b–e). The overexpression lines also maintained significantly greater fresh weight at both concentrations of ABA and NaCl and under 10 mM D-mannitol and 50 μM MeJA. Under 5 mM D-mannitol and 100 μM MeJA, fresh weight tended to be greater in the combined overexpression group, but the differences were not statistically significant (Figure 7f–i).

Collectively, the combined *PmCPS*-overexpressing group maintained greater absolute primary root length and fresh weight under the tested phytohormone and abiotic stress conditions, indicating a greater capacity to sustain growth during treatment. However, because these plants already exhibited a pronounced growth advantage under control conditions, the data alone do not demonstrate enhanced stress resistance.

### 2.8. Effects of PmCPS Overexpression on Endogenous Phytohormone Levels and Pathway-Related Gene Expression in A. thaliana

**Figure 8:**
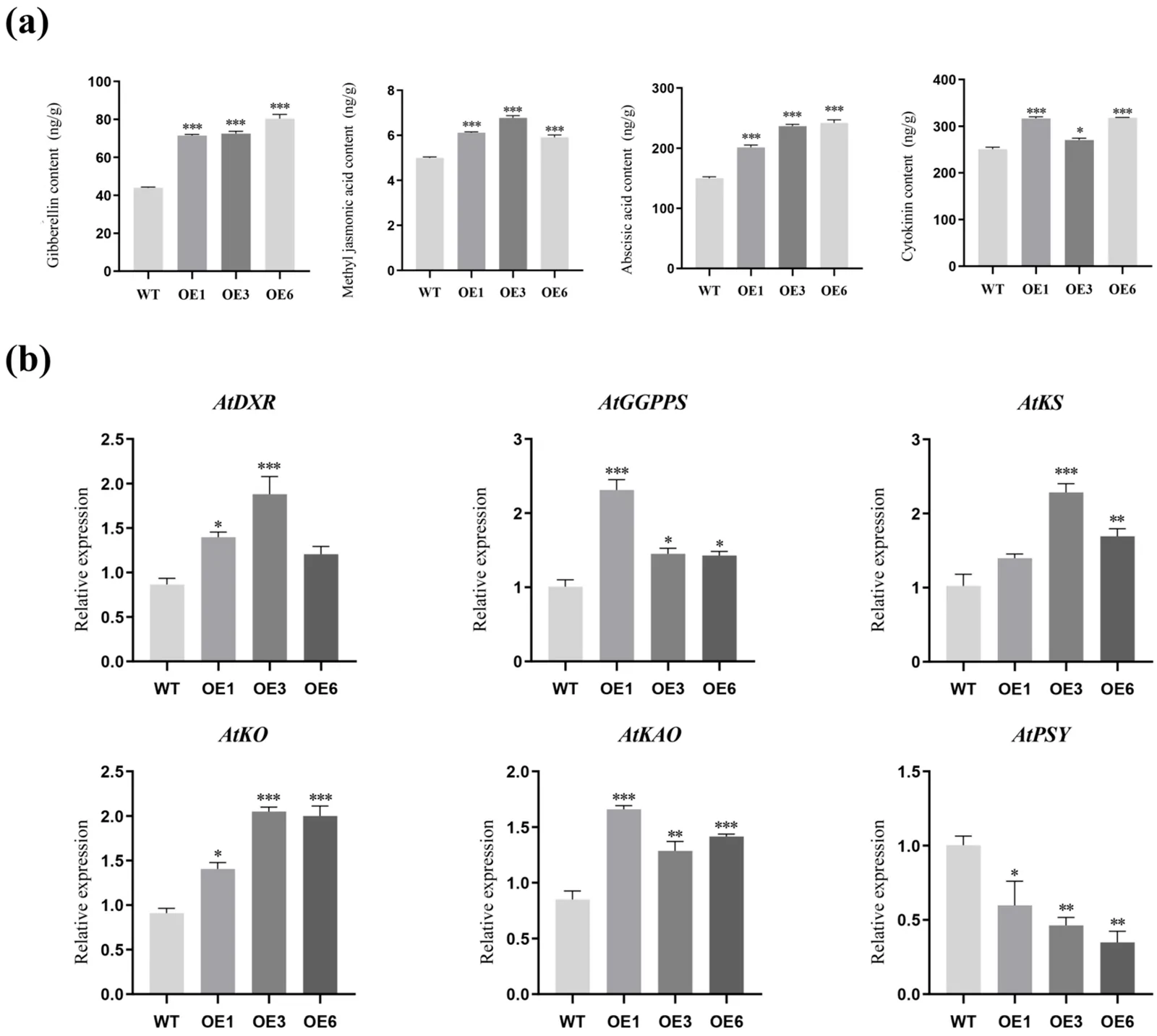
Effects of *PmCPS* overexpression on endogenous phytohormone levels and the expression of diterpenoid metabolism-related genes in *A. thaliana*. (**a**) GA_3_, MeJA, ABA, and CTK levels in wild-type plants and the OE1, OE3, and OE6 lines; (**b**) relative expression levels of *AtDXR*, *AtGGPPS*, *AtKS*, *AtKO*, *AtKAO*, and *AtPSY*. WT, wild type; OE1, OE3, and OE6, three independent *PmCPS*-overexpressing lines (* *p* < 0.0*5*; ** *p < 0.01*; *** *p* < 0.001; *n* = 3 biological replicates).

Endogenous levels of phytohormones associated with diterpenoid metabolism were measured in the transgenic *A. thaliana* lines. Among the phytohormones examined, GA_3_ showed the most pronounced increase (Figure 8a). Compared with the wild type (WT; 43.90 ± 0.67 ng g^−1^), GA_3_ contents in OE1, OE3, and OE6 reached 71.50, 72.44, and 80.39 ng g^−1^, respectively, and were significantly higher in all three lines. The increase in GA_3_ was consistent with the higher CPS activity and provided a plausible hormonal basis for the earlier bolting and greater biomass of the transgenic plants.

ABA, CTK, and MeJA contents were also higher in the overexpression lines than in WT plants. ABA increased significantly in all transgenic lines, with OE6 showing the largest increase (approximately 60% relative to WT). CTK levels were significantly higher in OE1 and OE6, whereas MeJA levels increased significantly in all three lines and were highest in OE3 (6.77 ± 0.18 ng g^−1^). Collectively, *PmCPS* overexpression increased CPS activity and GA_3_ accumulation and was accompanied by broader changes in phytohormone homeostasis. These hormonal changes may contribute to accelerated development and the maintenance of growth during treatment, although they do not independently demonstrate enhanced stress resistance.

To further investigate the effects of heterologous *PmCPS* expression on the endogenous metabolic network of *A. thaliana*, the transcript levels of key pathway-related genes were quantified by RT-qPCR.

The expression of several genes involved in terpenoid biosynthesis was significantly altered in the *PmCPS*-overexpressing lines (Figure 8b). *AtDXR*, which encodes a key rate-limiting enzyme in the MEP pathway, was significantly upregulated in OE1, OE3, and OE6, with the greatest increase observed in OE3. *AtGGPPS*, which contributes to terpenoid precursor biosynthesis, was also significantly upregulated, with the greatest increase observed in OE1. In the GA biosynthetic pathway, *AtKS*, *AtKO*, and *AtKAO* showed significantly higher expression in most overexpression lines than in WT plants; *AtKO* and *AtKAO* were particularly strongly upregulated in OE3 and OE6. By contrast, *AtPSY*, a key carotenoid biosynthesis gene, was significantly downregulated in all overexpression lines, suggesting an adjustment of the plastidial isoprenoid metabolic network. This expression pattern is consistent with coordinated feedback regulation and a potential shift in precursor allocation toward GAs and other diterpenoids; however, direct evidence of metabolic flux redistribution would require metabolomic or isotope-tracing analyses.

## 3. Discussion

### 3.1. Sequence Characteristics and Evolutionary Relationships

*P. massoniana* is an important timber- and oleoresin-producing tree species in southern China, and its growth, development, and environmental adaptation are closely associated with terpenoid metabolism [1,2,3,4,5,6]. Copalyl diphosphate synthase (CPS) catalyzes the cyclization of geranylgeranyl diphosphate (GGPP) to copalyl diphosphate (CPP) and is a key enzyme in the biosynthesis of gibberellins (GAs) and numerous diterpenoids [12,14,19,20,21,22]. Although the catalytic functions and evolutionary relationships of CPS proteins have been characterized in rice, *A. thaliana*, and several conifers, the regulation of CPS expression and its effects on the terpenoid metabolic network in *P. massoniana* remain poorly understood. Therefore, this study combined bioinformatics analyses, expression profiling, VIGS-mediated transient silencing in *P. massoniana*, and heterologous overexpression in *A. thaliana* to characterize *PmCPS* at the molecular, biochemical, physiological, and phenotypic levels.

PmCPS contains the characteristic Terpene_synth and Terpene_synth_C domains and retains the conserved motif required for class II diterpene cyclase activity. Phylogenetic analysis showed that PmCPS clustered most closely with CPS from *P. tabuliformis* and formed a gymnosperm-specific clade with CPS homologs from spruce and other gymnosperms, distinct from the angiosperm clade. Previous studies have shown that some bryophyte diterpene synthases retain bifunctional CPS/KS activity, whereas GA biosynthesis in seed plants is generally catalyzed sequentially by monofunctional CPS and KS enzymes. This evolutionary pattern reflects the functional divergence of plant diterpene synthases [19,22,26,27]. An induced protein band of approximately 83 kDa confirmed that recombinant PmCPS could be produced in *Escherichia coli*; however, its substrate specificity and catalytic products require direct verification using purified protein and in vitro enzymatic assays (Figure S2). The association network constructed using *A. thaliana* homologs suggested that CPS occupies an important position in the metabolic network, although this prediction does not demonstrate direct physical interactions between PmCPS and the associated proteins.

### 3.2. Tissue Expression and Hormonal Regulation

*PmCPS* was expressed in all tissues examined, with the highest transcript abundance detected in needles. The high expression of CPS and related diTPS genes in white spruce needles, together with the established role of terpenoid resins in constitutive and induced conifer defenses [3,4,5,21,30,31], provides a biological rationale for examining its possible roles in needle metabolism and defense. The present study did not quantify resin acids or test resistance to pathogens or herbivores; therefore, needle expression alone does not establish a direct chemical-defense function for *PmCPS*. At 24 h after MeJA treatment, *PmCPS* transcript abundance increased to approximately twice the pretreatment level. This response is consistent with reports that MeJA induces traumatic resin duct formation, terpene synthase expression, and terpenoid accumulation in spruce [32,33,34,35]. By contrast, GA_3_ treatment induced a transient early increase in *PmCPS* expression followed by a decline; this subsequent repression may reflect feedback regulation of GA homeostasis [12,13]. Nevertheless, predicted promoter cis-acting elements and transcriptional responses indicate only potential regulatory relationships; the upstream transcription factors and their corresponding binding sites require further experimental validation.

### 3.3. PmCPS Silencing and Gibberellin Homeostasis

VIGS-mediated silencing of *PmCPS* in *P. massoniana* achieved a mean silencing efficiency of 69% and significantly reduced CPS enzymatic activity. This reduction was accompanied by the general downregulation of *PmDXR*, *PmGGPPS*, *PmKS*, *PmKO*, and *PmKAO* and by a significant decrease in GA_3_ content. These findings are consistent with the biochemical position of CPS at an early committed step in GA biosynthesis and with the impaired GA production caused by loss of *SiDWARF4/CPS* function in foxtail millet [12,29]. Because *PmCPS* is not a transcription factor, the coordinated expression changes in upstream and downstream pathway genes are more likely to reflect altered metabolic flux, product feedback, or phytohormone crosstalk than direct transcriptional regulation by *PmCPS*. The silenced plants showed no obvious morphological changes during the observation period, possibly because of the limited duration of VIGS, the perennial growth habit of *P. massoniana*, and residual GA levels sufficient to sustain basal growth. Previous VIGS studies in woody plants have similarly shown that silencing duration and tissue-to-tissue spread can limit the manifestation of visible phenotypes [36].

In addition to GA_3_, MeJA and CTK levels decreased significantly following *PmCPS* silencing, whereas ABA showed a nonsignificant decreasing trend, indicating that disruption of *PmCPS* function was accompanied by broader changes in phytohormone homeostasis. GA is a diterpenoid phytohormone, and its reduction is directly consistent with the observed decrease in CPS activity. ABA is derived from carotenoid cleavage, whereas cytokinin side-chain biosynthesis involves isoprenoid precursors; both processes may therefore be affected by changes in plastidial isoprenoid metabolism. MeJA is derived from fatty acid oxidation and is not a direct catalytic product of *PmCPS*. Its altered abundance is more likely to result indirectly from growth–defense signaling crosstalk or metabolic feedback. Given the extensive bidirectional regulation between phytohormone signaling and metabolism [17,18,37], the present findings do not establish a direct causal relationship between *PmCPS* activity and MeJA accumulation.

### 3.4. Heterologous Overexpression and Growth Responses

Complementing the silencing results, heterologous overexpression of *PmCPS* significantly increased CPS activity and GA_3_ content in *A. thaliana* and was accompanied by larger leaves, greater fresh weight, and earlier bolting. These phenotypes are consistent with the established roles of GAs in promoting cell elongation and floral transition [12,13]. Previous studies have shown that enhanced GA biosynthesis in the secondary vascular tissues of poplar promotes cambial activity and xylem development [38], whereas altered *NtCPS2* expression in tobacco regulates glandular trichome development by affecting GA biosynthesis [39]. Under ABA, MeJA, NaCl, and D-mannitol treatments, the *PmCPS*-overexpressing lines maintained greater primary root length and fresh weight than wild-type plants, suggesting a greater capacity to sustain growth during treatment. However, because the transgenic lines were also larger under control conditions, these data do not demonstrate enhanced stress resistance per se.

### 3.5. Pathway Feedback and Study Limitations

Pathway-gene expression analysis showed that *AtDXR*, *AtGGPPS*, and most GA biosynthesis-related genes were upregulated following *PmCPS* overexpression, whereas *AtPSY* was downregulated. In the *P. massoniana* silencing materials, *PmPSY* was significantly downregulated only in the plant with the greatest silencing efficiency. Because the MEP pathway supplies precursors for GGPP and multiple classes of plastidial isoprenoids [16,17], two possible metabolic responses can be proposed. First, increased demand for CPP formation in *PmCPS*-overexpressing plants may stimulate upstream precursor supply while restricting the carotenoid branch through competition for GGPP. Second, following *PmCPS* silencing, pathway feedback may reduce overall precursor supply, with *PmPSY* expression being significantly affected only under severe perturbation. Because GGPP, CPP, carotenoids, and specific diterpenoid products were not directly quantified, these models remain hypothetical and require validation through targeted metabolomic analyses and in vitro enzymatic assays. Commercial ELISA measurements and pathway-gene expression changes provide indirect evidence of metabolic effects but do not identify the products formed by *PmCPS*. In addition, the combined OE1, OE3, and OE6 data in the growth assays do not resolve line-specific responses. Future work should characterize purified *PmCPS* products, analyze individual transgenic lines, and test defense-related metabolites and biotic challenges in *P. massoniana*.

## 4. Materials and Methods

### 4.1. Plant Materials, Bacterial Strains, and Vectors

Needles used to clone the *PmCPS* coding sequence and promoter were collected from two-year-old *P. massoniana* seedlings grown in a second-generation seed orchard at Baisha State-Owned Forest Farm, Shanghang County, Fujian Province, China. Materials for tissue-specific expression analysis were obtained from the pine wood nematode-resistant *P. massoniana* clone ‘Guang 50-4’, which was grafted in 2008 and maintained at the Shahe Forest Germplasm Resources Propagation Center of the Anhui Academy of Forestry. The selected trees were vigorous and showed no visible symptoms of disease or insect infestation. Needles, male strobili, female strobili, cones, young stems, mature stems, and roots were collected during the active growing season, immediately frozen in liquid nitrogen, and stored at −80 °C. Two-year-old *P. massoniana* seedlings grown at the State Key Laboratory of Tree Genetics and Breeding, Nanjing Forestry University, were used for phytohormone and abiotic stress treatments; one-year-old seedlings grown in the same laboratory were used for VIGS experiments.

The *A. thaliana* material used in this study was the Columbia-0 (Col-0) ecotype maintained at the State Key Laboratory of Tree Genetics and Breeding, Nanjing Forestry University. Plants were grown at 22 °C under a 16 h light/8 h dark photoperiod. Half-strength Murashige and Skoog (1/2 MS) medium contained 20 g L^−1^ sucrose and 6.5 g L^−1^ agar and was adjusted to pH 5.80 ± 0.05.

The pCE3-blunt cloning vector, FastPure Plant Total RNA Isolation Kit, FastPure Cell/Tissue DNA Isolation Mini Kit, FastPure Plasmid Mini Kit, and Gel DNA Extraction Mini Kit were purchased from Vazyme Biotech Co., Ltd. (Nanjing, China). The pTRV1, pTRV2, and pCAMBIA1302 vectors were maintained at the State Key Laboratory of Tree Genetics and Breeding, Nanjing Forestry University. Competent *Escherichia coli* DH5α cells were purchased from Kangti Biotechnology Co., Ltd. (Shenzhen, China), and *Agrobacterium tumefaciens* GV3101 was purchased from Weidi Biotechnology Co., Ltd. (Shanghai, China). Restriction enzymes (QuickCut™ NcoI, BglII) were obtained from Takara Biotechnology Co., Ltd. (Dalian, China). The Hifair^®^ III 1st Strand cDNA Synthesis Kit and Hieff UNICON Universal Blue qPCR SYBR Green Master Mix were purchased from Yeasen Biotech (Shanghai, China).

Chemical reagents and their sources were as follows: Methyl jasmonate (MeJA), salicylic acid (SA), D-mannitol, abscisic acid (ABA), NaCl, ethephon (ETH), gibberellin A_3_ (GA_3_), and hydrogen peroxide (H_2_O_2_) were obtained from Beijing Solarbio Science and Technology Co., Ltd. (Beijing, China). Hygromycin (Hyg), agar, 2-morpholinoethanesulfonic acid (MES), acetosyringone (AS), MgCl_2_, and Silwet L-77 were obtained from Nanjing Zebra Experimental Equipment Co., Ltd. (Nanjing, China). Kanamycin sulfate (Kan), ampicillin (Amp), and rifampicin (Rif) were obtained from Shanghai Yeasen Biotech (Shanghai, China). MS basal salts and 1/2 MS medium were obtained from Haixingli Biotechnology Co., Ltd. (Nanjing, China), and sucrose was obtained from Sangon Biotech (Shanghai, China).

### 4.2. Cloning and Prokaryotic Expression of PmCPS

Total RNA was extracted from *P. massoniana* needles using the FastPure Plant Total RNA Isolation Kit (RC401; Vazyme Biotech, Nanjing, China). RNA integrity was assessed by 1.2% agarose gel electrophoresis, and RNA concentration and purity were measured using a NanoDrop spectrophotometer (Thermo Fisher Scientific, Waltham, MA, USA). Genomic DNA was removed from 1 μg of total RNA, and first-strand cDNA was synthesized using the Hifair^®^ III 1st Strand cDNA Synthesis Kit (11139ES10; Yeasen Biotech, Shanghai, China). Primers were designed based on the *P. massoniana* reference genome sequence (Table S1). PCR products were purified using the FastPure Gel DNA Extraction Mini Kit (DC301-01; Vazyme Biotech, Nanjing, China) and ligated into pCE3-blunt using the Ultra-Universal TOPO Cloning Kit (C603-01; Vazyme Biotech, Nanjing, China). The ligation products were transformed into *Escherichia coli* DH5α, screened by colony PCR using M13-F/R primers, and subjected to Sanger sequencing. The resulting sequences were assembled and aligned using SnapGene v6.0.2. All primers were synthesized by Tsingke Biotechnology Co., Ltd. (Beijing, China) or Sangon Biotech Co., Ltd. (Shanghai, China).

For prokaryotic expression, the pET28a-MBP-6×His vector was linearized by inverse PCR, and the *PmCPS* coding sequence was amplified from cDNA using the MBP-*PmCPS*-His-F/R primers (Table S1). Homologous recombination was performed in a 10 μL reaction containing 210 ng of linearized vector, 50 ng of purified insert, 2 μL of 5× CE II Buffer, and 1 μL of Exnase II. The reaction mixture was incubated at 37 °C for 30 min and subsequently cooled on ice. Following cloning and sequence verification, the recombinant plasmid was extracted using the FastPure Plasmid Mini Kit and introduced into *Escherichia coli* Rosetta competent cells. Positive transformants were cultured in LB medium containing 50 mg L^−1^ ampicillin at 37 °C and 200 rpm until the OD600 reached approximately 0.6. Recombinant protein expression was induced with 0.3 mM isopropyl β-D-1-thiogalactopyranoside (IPTG) at 18 °C for 16 h. Cells were harvested by centrifugation at 6000 rpm and 4 °C for 5 min and resuspended in prechilled lysis buffer containing 50 mM NaH2PO4, 500 mM NaCl, 10 mM imidazole, and 1 mM phenylmethylsulfonyl fluoride (PMSF), pH 8.0. The suspension was sonicated on ice at 300 W using alternating 3 s pulses and 3 s intervals. Following centrifugation at 13,000 rpm and 4 °C for 10 min, the supernatant was collected and analyzed by SDS-PAGE using a 12% resolving gel and a 5% stacking gel. Electrophoresis was performed at 90 V for 10 min followed by 150 V for 40 min, and protein bands were visualized by Coomassie Brilliant Blue staining.

### 4.3. Bioinformatics and Phylogenetic Analyses of PmCPS

Conserved domains in the PmCPS protein were identified using the NCBI Conserved Domain Search tool (https://www.ncbi.nlm.nih.gov/Structure/cdd/wrpsb.cgi; accessed on 12 March 2025). Multiple sequence alignment was performed using MUSCLE (https://www.ebi.ac.uk/Tools/msa/muscle/; accessed on 12 March 2025), and the alignment was visualized using MSAVis (https://www.novopro.cn/tools/muscle.html; accessed on 12 March 2025).

Plant CPS homolog sequences were downloaded from the NCBI Protein database (https://www.ncbi.nlm.nih.gov/protein/; accessed on 12 March 2025). A phylogenetic tree was constructed in MEGA v11.0 using the neighbor-joining method with 1000 bootstrap replicates and was annotated and visualized using EvolView (https://www.evolgenius.info/evolview-v2/; accessed on 12 March 2025).

The molecular weight, theoretical isoelectric point, and amino acid composition of PmCPS were predicted using ExPASy ProtParam (https://web.expasy.org/protparam/; accessed on 12 March 2025), and protein hydropathy was analyzed using ExPASy ProtScale (https://web.expasy.org/protscale/; accessed on 12 March 2025). Signal peptides and transmembrane domains were predicted using SignalP v4.1 (https://services.healthtech.dtu.dk/services/SignalP-4.1/; accessed on 12 March 2025) and TMHMM v2.0 (https://services.healthtech.dtu.dk/services/TMHMM-2.0/; accessed on 12 March 2025), respectively. Secondary structure was predicted using SOPMA (https://npsa-prabi.ibcp.fr/cgi-bin/npsa_automat.pl?page=npsa_sopma.html; accessed on 12 March 2025), and three-dimensional homology modeling was performed using SWISS-MODEL (https://swissmodel.expasy.org/; accessed on 12 March 2025). Using the *Arabidopsis thaliana* CPS homolog as a reference, protein associations were predicted using STRING (https://string-db.org/; accessed on 12 March 2025), and the resulting network was visualized in Cytoscape (v3.9.1, https://cytoscape.org/; accessed on 12 March 2025).

### 4.4. Cloning of the PmCPS Promoter and Analysis of Cis-Acting Elements

Genomic DNA was extracted from *P. massoniana* needles using the FastPure Plant DNA Isolation Mini Kit (DC104-01; Vazyme Biotech, Nanjing, China). Specific primers designed from the *P. massoniana* reference genome [40] were used to amplify the sequence upstream of *PmCPS* (Table S1). The PCR product was ligated into pCE3-blunt, cloned, and subjected to Sanger sequencing. The resulting sequence was assembled and aligned using SnapGene v6.0.2. Promoter cis-acting elements were predicted using PlantCARE and visualized using TBtools v2.376 [41].

### 4.5. Tissue-Specific Expression of PmCPS and Phytohormone and Abiotic Stress Treatments

Total RNA was extracted from needles, male strobili, female strobili, cones, young stems, mature stems, and roots of the pine wood nematode-resistant *P. massoniana* clone to analyze the tissue-specific expression pattern of *PmCPS*. Three biological replicates were prepared for each tissue, and female strobili were used as the calibrator for relative expression analysis.

Vigorous, uniformly sized two-year-old *P. massoniana* seedlings without visible symptoms of disease or insect infestation were selected for phytohormone and abiotic stress treatments. Treatment concentrations were determined from preliminary gradient experiments and previous studies [8,32,33,34,35]. Seedlings were subjected to foliar application of 10 mM H_2_O_2_, 50 μM ethephon (ETH), 100 μM ABA, 1 mM SA, 2 mM GA_3_, or 10 mM MeJA. For salt treatment, each pot (containing approximately 500 g of soil) was irrigated with 200 mL of 200 mM NaCl solution in a single application. The rhizosphere soil was thoroughly irrigated with 200 mM NaCl. Needles were collected at 0, 3, 6, 12, and 24 h after treatment, immediately frozen in liquid nitrogen, and stored at −80 °C. Three biological replicates were prepared for each treatment and time point, with three technical replicates per biological replicate. The corresponding 0 h samples collected before treatment were used as calibrators for relative expression analysis.

### 4.6. Quantitative Real-Time PCR Analysis

Primers for quantitative real-time PCR (RT-qPCR) were designed using Primer Premier v5.0 and Primer-BLAST (https://www.ncbi.nlm.nih.gov/tools/primer-blast/; accessed on 12 March 2025) and are listed in Table S1. RT-qPCR was performed using an ABI StepOnePlus Real-Time PCR System. Each 10 μL reaction contained 1 μL of cDNA, 0.4 μL each of the forward and reverse primers, 5 μL of Hieff UNICON Universal Blue qPCR SYBR Green Master Mix (11184ES03; Yeasen Biotech, Shanghai, China), and 3.2 μL of nuclease-free water.

The amplification program consisted of an initial denaturation at 95 °C for 2 min, followed by 40 cycles of denaturation at 95 °C for 10 s and annealing and extension at 60 °C for 30 s. The α-tubulin gene *TUA* (GenBank accession no. KM496535.1) was used as the reference gene for *P. massoniana*, as in Wang et al. [42]. *AtActin* (*ACTIN2*/*ACT2*; AT3G18780) was used as the reference gene for *A. thaliana*, consistent with its previous use by Tanvir et al. [43] (primer sequences are listed in Table S1). Three biological replicates were analyzed for each treatment or genotype, with three technical replicates per biological replicate. Relative expression was calculated using the 2^^−ΔΔCt^ method [44]. ΔCt was defined as Ct(target) − Ct(reference), and ΔΔCt as ΔCt(sample) − ΔCt(calibrator). Technical replicate Ct values were averaged for each biological sample before calculating relative expression. Female strobili, pretreatment 0 h samples, empty-vector controls, or wild-type plants served as the respective calibrators for the different experiments.

### 4.7. VIGS-Mediated Silencing of PmCPS

Primers targeting the *PmCPS* fragment used for VIGS were designed using Primer Premier v5.0 (Table S1). Homologous arms corresponding to the *Eco*RI and *Bam*HI sites in pTRV2 were added to the 5′ ends of the primers, and the pTRV2-*PmCPS* construct was generated by homologous recombination [42]. After sequence verification, the recombinant plasmid was transformed into *Agrobacterium tumefaciens* GV3101.

*Agrobacterium* cultures harboring pTRV1, pTRV2, or pTRV2-*PmCPS* were grown separately in LB liquid medium containing 50 mg L^−1^ kanamycin and 25 mg L^−1^ rifampicin at 28 °C and 200 rpm until the OD_600_ reached approximately 1.0. Cells were harvested by centrifugation at 6000 rpm for 1 min and resuspended in infiltration buffer containing 10 mM MES, 10 mM MgCl_2_, and 200 μM acetosyringone (pH 5.6). The pTRV1 suspension was mixed with either the pTRV2 or pTRV2-*PmCPS* suspension at a 1:1 volume ratio. The final OD_600_ was adjusted to 1.0, and the mixtures were incubated at room temperature in darkness for 3 h. Silwet L-77 was then added to a final concentration of 0.1%.

Uniformly growing one-year-old *P. massoniana* seedlings were selected, and soil was carefully removed from the roots. The stems and needles were completely immersed in the infiltration suspension and vacuum-infiltrated at −0.08 MPa for 5 min. After the pressure was slowly returned to atmospheric pressure, excess bacterial suspension was removed from the plant surfaces with sterile filter paper. The seedlings were transplanted into growth substrate and maintained in darkness overnight before transfer to a growth chamber. A second infiltration was performed 7 d after the first, followed by a third infiltration 3 d later. Seedlings treated with pTRV1 + pTRV2 served as empty-vector controls.

Needles were collected after the final infiltration. *PmCPS* transcript abundance was determined by RT-qPCR, and *PmCPS*, pTRV1, and pTRV2 fragments were detected by semi-quantitative PCR, with *TUA* used as the reference gene. Plants with high silencing efficiencies were selected and designated pTRV2-1, pTRV2-5, and pTRV2-6 for subsequent analyses. For each treatment, three biological replicates were analyzed, with three technical replicates per biological replicate.

### 4.8. Analysis of CPS Enzymatic Activity, Endogenous Phytohormones, and Pathway-Related Gene Expression

For the *P. massoniana* experiment, *PmCPS*-silenced plants validated by RT-qPCR were selected, and plants carrying the pTRV1 + pTRV2 empty vectors served as controls. Needles were collected from equivalent positions on each plant. For the *A. thaliana* experiment, four-week-old wild-type plants and the T_3_ OE1, OE3, and OE6 lines were used, and the third to fifth true leaves were collected. For each genotype, equal amounts of leaf tissue from three plants were pooled to form one biological replicate, and three biological replicates were prepared. All samples were frozen immediately in liquid nitrogen.

CPS enzymatic activity and GA_3_, MeJA, ABA, and CTK levels were determined using commercial ELISA kits (Shanghai Huding Biotechnology Co., Ltd., Shanghai, China) according to the manufacturer’s instructions. The kits used were as follows: ent-Copalyl Diphosphate Synthase (ent-CDPS) Activity ELISA Kit (DS419X-Pt), Gibberellin A_3_ (GA_3_) ELISA Kit (DS263-Pt), Abscisic Acid (ABA) ELISA Kit (DS087-Pt), Methyl Jasmonate (MeJA) ELISA Kit (DS394-Pt), and Cytokinin (CTK) ELISA Kit (DS065-Pt). All kits employed a double-antibody sandwich method. Briefly, samples were added to microplate wells pre-coated with the corresponding primary antibody, followed by the addition of HRP-conjugated secondary antibody. After washing, TMB substrate was added for color development, and the reaction was terminated with stop solution. Absorbance was measured at 450 nm using a microplate reader.

Relative expression levels of pathway-related genes were measured by RT-qPCR. In *P. massoniana*, the MEP pathway and precursor-supply genes *PmDXR* and *PmGGPPS*, the GA biosynthesis genes *PmKS*, *PmKO*, and *PmKAO*, and the carotenoid biosynthesis gene *PmPSY* were analyzed, with *TUA* used as the reference gene. In *A. thaliana*, *AtDXR*, *AtGGPPS*, *AtKS*, *AtKO*, *AtKAO*, and *AtPSY* were analyzed, with *AtActin* used as the reference gene. For each *A. thaliana* line, 24 seedlings were selected, and equal amounts of tissue from eight seedlings were pooled to form one biological replicate, yielding three biological replicates. All RT-qPCR assays included three technical replicates per biological replicate. Primer sequences are listed in Table S1.

### 4.9. Generation and Identification of PmCPS-Overexpressing A. thaliana

The *PmCPS* coding sequence was inserted into pCAMBIA1302 by homologous recombination to generate the pCAMBIA1302-*PmCPS* overexpression construct. After sequence verification, the recombinant plasmid was transformed into *A. tumefaciens* GV3101. Genetic transformation, antibiotic-resistance screening, and isolation of homozygous *A. thaliana* lines were performed as described by Wang et al. [42]. Transgenic plants were identified by genomic PCR and RT-qPCR, and the homozygous T_3_ lines OE1, OE3, and OE6 were selected for subsequent experiments.

### 4.10. Growth Phenotyping and Treatment Responses of Transgenic A. thaliana

Seeds of the homozygous T_3_ OE1, OE3, and OE6 lines and wild-type *A. thaliana* were surface-sterilized and sown on 1/2 MS medium. After stratification in darkness at 4 °C for 48 h, the seeds were transferred to 22 °C under a 16 h light/8 h dark photoperiod. Primary root length and fresh weight were measured after 10 d of growth, and seedling phenotypes were recorded. A separate set of uniformly growing seedlings was transplanted into nutrient soil and grown for an additional 20 d, after which plant phenotypes, fresh weight, and bolting time were recorded. For the growth comparisons in Figure 6b–d, observations from OE1, OE3, and OE6 were combined into the *PmCPS* overexpression group.

For the treatment experiments, 1/2 MS medium was supplemented with 0.4 or 0.6 μM ABA, 50 or 100 μM MeJA, 100 or 150 mM NaCl, or 5 or 10 mM D-mannitol. Seeds of wild-type and *PmCPS*-overexpressing plants were surface-sterilized, stratified at 4 °C for 48 h, and sown on untreated medium or medium containing the corresponding treatment compound. Plates were incubated vertically. After 14 d, seedling phenotypes were photographed, and primary root length and fresh weight were measured. Thirty seedlings were analyzed for each genotype and treatment, and the experiment was independently repeated three times.

### 4.11. Statistical Analysis

Data are presented as the mean ± standard error (SE). The mean of the technical replicates was used as the value for each biological replicate; technical replicates were not treated as independent samples in statistical analyses. Statistical analyses were performed using IBM SPSS Statistics v25, and graphs were generated using GraphPad Prism v8.

Comparisons among tissue types, treatment time points, silenced plants, and multiple overexpression lines were performed using one-way analysis of variance followed by Duncan’s multiple range test. CPS enzymatic activity and phytohormone data involving two independent groups were analyzed using two-tailed independent-samples *t*-tests. Differences were considered significant at *p* < 0.05. In the figures, *, **, ***, and **** indicate *p* < 0.05, *p* < 0.01, *p* < 0.001, and *p* < 0.0001, respectively.

## 5. Conclusions

In this study, the diterpenoid biosynthesis-related gene *PmCPS* was cloned and characterized from *P. massoniana*. The encoded protein exhibited the characteristic structural features of a class II diterpene cyclase and was highly conserved among gymnosperm CPS homologs. *PmCPS* transcript abundance was highest in needles and increased transiently after GA_3_ treatment. VIGS-mediated silencing reduced *PmCPS* transcript abundance and CPS enzymatic activity, decreased GA_3_, MeJA, and CTK levels, and suppressed genes associated with the MEP pathway and GA biosynthesis. Conversely, heterologous overexpression of *PmCPS* in *A. thaliana* increased CPS activity and GA_3_ content, promoted biomass accumulation, accelerated bolting, and enhanced the capacity of plants to maintain growth under the tested treatment conditions. Together, the complementary silencing and overexpression results indicate that *PmCPS* is an important component of GA biosynthesis and diterpenoid metabolism in *P. massoniana* and may contribute to plant growth and treatment responses through interactions between metabolism and phytohormone signaling. This study identifies a candidate gene for elucidating the regulation of diterpenoid metabolism in conifers and for genetically improving economically and adaptively important traits in *P. massoniana*.

## Data Availability

The original contributions presented in this study are included in the article/Supplementary Material. Further inquiries can be directed to the corresponding author.

## Supplementary Materials

The following supporting information can be downloaded at: https://www.mdpi.com/article/10.3390/plants15213273/s1, Figure S1, cloning of the *PmCPS* open reading frame; Figure S2, Prokaryotic Expression of PmCPS Protein; Table S1. List of the 21 PmWOX genes identified in this study; Table S2. Primers used in this study; Table S3. Reference WOX genes from *A. thaliana* and *P. tabuliformis* in this study.
