# Supplementary material for: Cloning and Functional Characterization of PmCPS, a Copalyl Diphosphate Synthase Gene from Pinus massoniana Lamb
Source: Plants (Basel). 2026 Oct 23;15(21):3273. doi: 10.3390/plants15213273 (PMC13765750; doi:10.3390/plants15213273)
Supplement: Supplementary file 1 [file plants-15-03273-s001.zip › Supplementary Figure.pdf]

Supplementary Figure S1

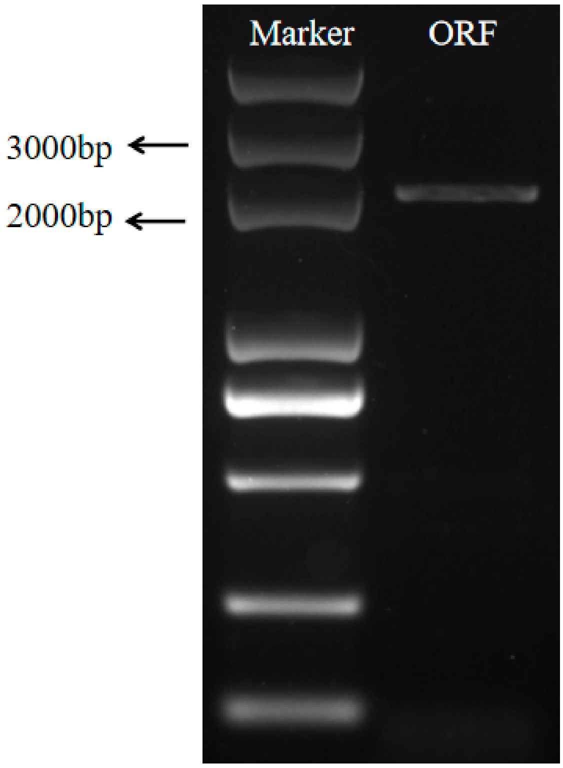

Figure S1. Cloning of the *PmCPS* open reading frame

Supplementary Figure S2

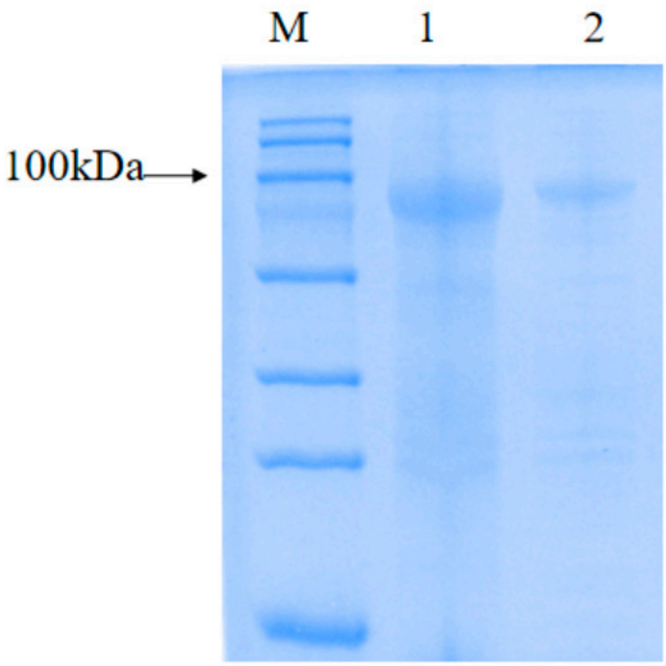

Figure S2. Prokaryotic Expression of PmCPS Protein
